# Utilization of patient portals: a cross-sectional study investigating associations with mobile app quality

**DOI:** 10.1186/s12911-023-02252-x

**Published:** 2023-09-05

**Authors:** Noha El Yaman, Jad Zeitoun, Rawan Diab, Mohamad Mdaihly, Razan Diab, Lynn Kobeissi, Salwa Abou Ljoud, Jumana Antoun, Marco Bardus

**Affiliations:** 1 Faculty of Medicine, American University of Beirut, Beirut, Lebanon; 2 Department of Family Medicine, Faculty of Medicine, American University of Beirut, Beirut, Lebanon; 3 Institute of Applied Health Research, College of Medical and Dental Sciences, University of Birmingham, Birmingham, B15 2TT UK; 4 Department of Health Promotion and Community Health, Faculty of Health Sciences, American University of Beirut, Beirut, Lebanon

**Keywords:** Patient portals, Electronic Health Record, Usability, Usage frequency, mHealth, Middle East, Lebanon

## Abstract

**Background:**

Mobile apps facilitate patients’ access to portals and interaction with their healthcare providers. The COVID-19 pandemic accelerated this trend globally, but little evidence exists on patient portal usage in the Middle East, where internet access and digital literacy are limited. Our study aimed to explore how users utilize a patient portal through its related mobile app (MyChart by EPIC).

**Methods:**

We conducted a cross-sectional survey of MyChart users, recruited from a tertiary care center in Lebanon. We collected MyChart usage patterns, perceived outcomes, and app quality, based on the Mobile Application Rating Scale (user version, uMARS), and sociodemographic factors. We examined associations between app usage, app quality, and sociodemographic factors using Pearson’s correlations, Chi-square, ANOVA, and t-tests.

**Results:**

428 users completed the survey; they were primarily female (63%), aged 41.3 ± 15.6 years, with a higher education level (87%) and a relatively high crowding index of 1.4 ± 0.6. Most of the sample was in good and very good health (78%) and had no chronic illnesses (67%), and accessed the portal through MyChart once a month or less (76%). The most frequently used features were accessing health records (98%), scheduling appointments (67%), and messaging physicians (56%). According to uMARS completers (n = 200), the objective quality score was 3.8 ± 0.5, and the subjective quality was 3.6 ± 0.7. No significant association was found between overall app usage and the mobile app quality measured via uMARS. Moreover, app use frequency was negatively associated with education, socioeconomic status, and perceived health status. On the other hand, app use was positively related to having chronic conditions, the number of physician visits and subjective app quality.

**Conclusion:**

The patient portal usage was not associated with app quality but with some of the participants’ demographic factors. The app offers a user-friendly, good-quality interface to patient health records and physicians, appreciated chiefly by users with relatively low socioeconomic status and education. While this is encouraging, more research is needed to capture the usage patterns and perceptions of male patients and those with even lower education and socioeconomic status, to make patient portals more inclusive.

**Supplementary Information:**

The online version contains supplementary material available at 10.1186/s12911-023-02252-x.

## Background

Patient portals are being extensively studied as an empowering tool that securely connects patients to their physicians and allows them to access their health information to emphasize active patient engagement [1–3]. By using electronic patient portals through mobile apps, patients can access their health records, communicate asynchronously with their physicians, schedule appointments and check out health information related to their condition [2, 4–7]. Mobile apps have become an essential aspect of patient portals which integrate telemedical features to facilitate patient-physician interaction within hospital settings [2, 3, 8–11]. Patient portal use positively affects patient engagement [1, 5, 9, 12], health outcomes [2, 7, 12], and provider-patient communication [9, 12] [9, 12]. Furthermore, many patients reported increased self-efficacy and empowerment as they became partners in their care plan and decision-making [5, 13]. Some studies also claimed that patients’ ability to access their health information and test results at their convenience significantly facilitated future in-person patient-doctor communication [5].

Several studies examined usage patterns of patient portals and identified some predictors of patient portal utilization. For example, higher use of patient portals was reported among patients living in cities compared to residents of rural areas, especially among those distant from the care provider; lower age, low socioeconomic status, and having chronic conditions were also positively related to patient portal access [14–16]. Patients with chronic disease have lots of needs including more frequent hospital visits, communication with providers and pharmacists regarding their chronic medications, laboratory tests, and inpatient admissions. As such, patient portals provide patients with chronic conditions with the right tool for better communication with the healthcare system, increased medication adherence, and reduced travel time and time off from work [17]. Other studies suggested increased use when physicians encouraged it [18, 19]. Trust in a physician has been shown to increase adherence to the physician’s advice [20]. Moreover, some studies showed how usability elements (i.e., user experience, perceived usefulness, and ease of use) might be crucial to increase adherence to patient portals [4]. However, more research is needed to understand the relationship between usage and perceived usability of patient portals through an easy and accessible interface such as a mobile app.

Another critical factor that plays a role in accessing a patient portal is access to technology and internet connectivity. The so-called “digital divide”, which refers to the gap among different demographics and regions in access to information technology, is essential in adopting patient portals. For example, the speed of the internet is a crucial factor affecting patient portal use. In addition, the fear of information breaches and lack of confidentiality are common concerns that need to be addressed [21, 22]. Despite numerous efforts to improve access to health information, difficulties in navigation as displaying the health data in small-format and complex interfaces still create a gap in understanding the health data [23]. It has been shown that smaller smartphone screen size and smaller font size may decrease use of health apps on smartphones [24, 25]. Furthermore, literacy challenges, basic and routine computer barriers related to using a search bar or navigating a website, and difficulties understanding medical terminology create a burden in using patient portals [26]. It is important to address these challenges as previous studies have mentioned the clinically significant benefits of utilizing such portals on health outcomes such as blood pressure, Hba1c and LDL levels [27, 28]. From a wider health systems perspective, increased patient portal utilization is of particular interest as it leads to decreased per capita cost of healthcare with fewer no-show appointments, emergency room visits and preventable hospital stays [29]. A recent study on the level of eHealth literacy in Lebanon [30] showed that socioeconomic status, gender, and education were positively related to eHealth literacy. eHealth literacy has been shown to be positively associated with increased portal usage [24, 31, 32]. To develop personalized and targeted interventions aimed at increasing adoption and utilization of the portals, it is useful to understand the profile of the patients who might be disadvantaged. Furthermore, a study conducted among diabetic patients in Saudi Arabia showed that essential factors such as internet access, content, and language might be substantial barriers to patient portal utilization [10, 33].

While most of the evidence on patient portals comes from the Western world, little is known about the usage of such technology in developing countries, especially in the Middle East, a region that lags in the accelerated era of health informatics applications and electronic patient portals (EPP) [6, 34–36]. Even in some affluent countries such as the United Arab Emirates, the adoption and utilization of eHealth platforms are in their early stages, requiring governments to put extra effort into encouraging user engagement and raising awareness about the benefits of EPPs [37]. In Lebanon, recent reports suggest high penetration rates of internet [38], and widespread smartphones use [39]. In addition, a previous study on user acceptance of patient portals in Lebanon reported that about half of the sampled population reported a significant intent to use EPPs [33]. Given that the intention to use was lower than that reported in the Western literature, the authors feared even lesser actual app utilization rates [33, 40]. To our knowledge, studies on the usability, benefits and barriers to use of patient portals in developing countries are still scarce [12, 41–43]. Also, no studies from the Middle East or Arab world have examined the role of MyChart as a mobile app in providing access to patient portals.

This study aimed to evaluate how patients access, utilize, and perceive the quality of a tertiary care center patient portal that is accessible through a mobile app named MyChart. This study specifically examined the frequency of app use, features used, factors facilitating and hampering the usage of the portal, and the associations between portal use, sociodemographic characteristics, and perceived app quality.

## Methods

### Participants and procedures

This cross-sectional study was conducted at the American University of Beirut Medical Center (AUBMC), which offers a patient portal called MyChart (powered by EPIC). The study received ethical approval from the Institutional Review Board (ref #SBS-2021-0433). Between February and May 2022, MyChart users were invited to complete an anonymous web-based survey hosted on secure institution servers.

A link to the survey was circulated through the MyChart app notifications and emails among adult users who agreed to participate in research studies (the number of users was not disclosed by the Institution for ethical reasons, hence a response rate cannot be calculated). In addition, the same link was made available to patients in waiting areas in different hospital outpatient clinics. The link led to a consent form page, so all participants consented before starting the questionnaire. All MyChart users older than 18 years were eligible to participate. The questionnaire was available in English and Arabic.

### Measures

The questionnaire included three parts: (a) sociodemographic information, (b) app usage and its specific features along with factors influencing the usage, and (c) evaluation of the app quality, using the user version of the Mobile App Rating Scale (uMARS). The research team developed the questions tackling sociodemographic information and app usage based on previous studies; these were then translated into Arabic. The first two parts of the survey were translated into Arabic by two team members who are bilingual with native proficiency in the Arabic language and fluent in English. After the translation, the Arabic version was verified by our principal investigator. For the uMARS, we used the translated version developed through a process of translation, back-translation, and cultural adaptation of the scale into Arabic, as reported in a study by Bardus et al. [44].

The sociodemographic section included questions about age, gender, educational level, household crowding index (HCI) as a proxy indicator of socioeconomic status [45], as well as area of primary residence, perceived health status, presence of chronic illness, and number of physician visits before and after the COVID-19 pandemic.

One question operationalized app usage as follows: “On average, how many times did you use the MyChart app on your phone in the past year?”, with five ordinal categories ranging from once a week to once a year. If the participants responded positively, we asked follow-up questions about the specific features utilized (i.e., consulting health records/test results, scheduling in-person appointments, messaging physicians, consulting the patient education section, and participating in a remote visit). We also enquired about the reasons for using or not using such features.

Finally, we enquired about the perceived impact of the MyChart app through three items, assessed on a 5-point scale (range: to a very large extent-to a very little extent) such as “improving access to health information”, “improving communication with the physician”, and “feeling in control of your health”.

Perceived app quality was assessed through the user version of the Mobile App Rating Scale (uMARS) [46, 47]. The original expert-oriented version MARS has been adopted to assess the quality of mobile apps in different health contexts, such as mindfulness and mental health [46, 48], weight management [44], smartphone addiction [49], and many more. The MARS has demonstrated excellent interrater reliability (2-way mixed ICC = 0.79, 95% CI 0.75–0.83), internal consistency (Cronbach alpha = 0.90); additionally, the MARS score was significantly correlated with the 5-star rating item in the subjective quality scale, similar to the 5-star rating in the app stores, as a proxy indicator of validity [46]. Similar to the MARS [46], the uMARS is a simple, multi-dimensional, and reliable tool to evaluate the quality of mobile health apps and is one of the most utilized instruments to assess app quality currently available [50]. The uMARS score encompasses four “objective” domains, engagement, functionality, aesthetics, information quality, and one “subjective” domain [47]. The engagement, functionality, aesthetics, and information quality sections are respectively composed of 5, 4, 3, and 4 items each, while the subjective section consists of 4 items. All uMARS items are scored via 5-point Likert-type scales, and a score for each objective domain is calculated as the average of the individual items. A total uMARS score is calculated as the mean of the scores of all sub-domains [47, 50]. The subjective quality score is calculated as the average of the four items [46, 48, 44, 49].

Like the MARS, the uMARS has been utilized to evaluate the quality for several smartphone health apps [51–53]. The total uMARS score has excellent internal consistency (Cronbach alpha = 0.90) and good test-retest reliability (ICCs between 0.66 and 0.70 over two and three months) [47]. However, it has never been used to evaluate the quality of MyChart or other apps used to access patient portals. The English and Arabic versions of the uMARS which aimed to evaluate app quality were adopted from the studies by Stoyanov et al. and Bardus et al. respectively [44, 46]. The Arabic version of the MARS scale was highly correlated with the respective subscales in the English version [48]. The subjective quality scores and the 5-star rating were also highly correlated with the total MARS score [48] indicating a good level of validity. The questionnaire is included as a Supplementary Material.

### Statistical analyses

The data were analyzed using IBM SPSS Statistics (Version 27). Descriptive analysis of the demographics, overall app, features usage, facilitators, and barriers for the usage and non-usage of various features, as well as the uMARS items was performed using means and standard deviations for continuous variables and proportions for categorical variables. Participants who completed all the uMARS items were included in the analyses. Missing values were not imputed.

Under the assumption that the data follows a normal distribution, inferential analyses were performed to assess the association among the overall app usage frequency (primary outcome, categorical variable with > 2 categories) and the following variables: the specific features used (categorical variable with 2 categories), age (continuous), gender (categorical variable with 2 categories), education (categorical variable with 2 categories), crowding index as a proxy for socioeconomic status (continuous), perceived health status (categorical variable with > 2 categories), and uMARS scores (continuous). We used one-way ANOVA tests to establish associations between continuous variables and categorical variables with > 2 categories, Chi-squared tests for associations between 2 categorical variables and independent t-tests for associations between continuous variables and categorical variables with only 2 categories. Pearson’s correlation tests were performed to study the association between continuous variables (number of physician visits, uMARS total score and subjective quality score) and independent t-test for associations between continuous variables and categorical variables with only 2 categories (uMARS completers versus non-completers). Statistical significance was set at alpha < 0.05. For each ANOVA test, assuming independency of cases, we checked the assumptions of normality (normal probability plots), and homogeneity of variance (Levene’s test). If data violated one of the assumptions, we reported the F-test estimated through Brown–Forsythe test, as available in SPSS.

## Results

### Sample characteristics

A total of 428 users were recruited and completed the survey. Table 1 includes the characteristics of the whole sample. Overall, respondents were on average 41.3 ± 15.6 years, mostly females (268/424, 63.2%) and with a higher education level (368/422, 87.2%), with a relatively high socioeconomic status, with an average crowding index of 1.4 ± 0.6. Most respondents lived in the capital city of Beirut, where the tertiary center is located (260/415, 62.7%). Most of the participants perceived their health as good (173/424, 40.8%) or very good (156/424, 36.8%); one-third reported having chronic illnesses (138/419, 32.9%). The average number of physician visits per year before (2.9 ± 3.3) and after the COVID-19 pandemic (2.9 ± 3.2) was not significantly different (t=-0.881, P = 0.379).

Table 1 also compares the sub-sample of those who completed all app quality items (n = 200, “uMARS completers”), compared to those who provided insufficient information (n = 228, “uMARS non-completers”). On average, uMARS completers were relatively younger (p < 0.001) and had a significantly higher crowding index (p = 0.002), and were less likely to report chronic illnesses (p = 0.020) compared to uMARS non-completers.

**Table 1:** Characteristics of the sample, uMARS completers and non-completers

Variables	N	Total Sample	uMARS completers (n = 200)	uMARS non-completers (n = 228)	P value*
		M(SD) [range]	M(SD) [range]	M(SD) [range]	
Age	417	41.3 (15.6) [18–84]	36.8 (13.8) [18–84]	45.3 (16.0) [18–84]	< 0.001
Crowding Index	404	1.4 (0.6) [0.3-3.0]	1.5 (0.6) [0.25-3.0]	1.3 (0.5) [0.33–2.67]	0.002
Number of physician visits/year before the COVID-19 pandemic	417	2.9 (3.3) [0–30]	2.8 (2.5) [0–24]	3.1 (3.8) [0–33]	0.305
Number of physician visits/year after the COVID-19 pandemic	410	2.9 (3.2) [0–50]	2.7 (2.0) [0–10]	3.0 (4.0) [0–50]	0.332
Gender	424	n(%)	n(%)	n(%)	0.765
Female		268 (63.2)	126 (64.0)	142 (62.6)	
Male		156 (36.8)	71 (36.0)	85 (37.4)	
Highest education level attained)	422				0.349
Higher education		368 (87.2)	175 (88.8)	193 (85.8)	
Lower education		54 (12.8)	22 (11.2)	32 (14.2)	
Area of primary residence	415				0.165
Beirut		260 (62.7)	128 (65.3)	132 (60.3)	
Bekaa		3 (0.7)	0 (0)	3 (1.4)	
Mount Lebanon		122 (29.4)	51 (26.0)	71 (32.4)	
North		19 (4.6)	12 (6.1)	7 (3.1)	
South		11 (2.7)	5 (2.6)	6 (2.7)	
Perceived health status	424				0.360
Poor		8 (1.9)	2 (1.0)	6 (2.7)	
Fair		39 (9.2)	14 (7.1)	25 (11.1)	
Good		173 (40.8)	85 (42.9)	88 (38.9)	
Very Good		156 (36.8)	72 (36.4)	84 (37.2)	
Excellent		48 (11.3)	25 (12.6)	23 (10.2)	
Chronic illnesses	419				0.020
Present		138 (32.9)	54 (27.3)	84 (38.0)	
Absent		281 (67.1)	144 (72.7)	137 (62.0)	

* P value of Chi-square, ANOVA, or t-tests

### Patient portal usage

Table 2 presents information about the frequency of app use, features used, and perceived outcome of using the patient portal. About half of the respondents (226/428, 52.8%) reported accessing the patient portal through the MyChart app at least once a month. The most frequently used feature was consulting their health records (415/422, 98.3%), and the least used was participating in a remote visit (33/424, 7.8%). On average they used, 2.4 ± 1.0 out of the 5 features mentioned in the survey. uMARS completers were more likely to participate in a remote visit and use more features than non-completers (p = 0.018), and reported using more features (p = 0.030) than non-completers.

**Table 2:** App Usage Patterns of MyChart Users

Variables	N	Total Sample	uMARS completers (n = 200)	uMARS non- completers (n = 228)	P value*
		n(%)	n(%)	n(%)	
Frequency of app use	428				0.607
More than once a week		43 (10.0)	16 (8.0)	27 (11.8)	
Once a week		62 (14.5)	33 (16.5)	29 (12.7)	
Once a month		121 (28.3)	56 (28.0)	65 (28.5)	
Once every few months		182 (42.5)	85 (42.5)	97 (42.5)	
Once a year		20 (4.7)	10 (5.0)	10 (4.4)	
Features used					
Consulting health records/test results	422	415 (98.3)	197 (99.0)	218 (97.8)	0.321
Scheduling in-person appointments	425	283 (66.6)	131 (65.5)	152 (67.6)	0.654
Messaging physicians	419	233 (55.6)	116 (58.3)	117 (53.2)	0.293
Consulting the patient education section	417	82 (19.2)	46 (23.2)	36 (16.4)	0.081
Participating in a remote visit	424	33 (7.8)	22 (11.1)	11 (4.9)	0.018
		M(SD) [range]	M(SD) [range]	M(SD) [range]	
Average number of features used	427	2.4 (1.0) [0–5]	2.6 (1.0) [1–5]	2.3 (1.0) [0–5]	0.030
Perceived impact of the app					
Improving access to health information	408	3.7 (1.0) [1–5]	3.8 (0.9) [1–5]	3.7 (1.0) [1–5]	0.178
Improving communication with physician	396	3.0 (1.2) [1–5]	3.1 (1.1) [1–5]	3.0 (1.2) [1–5]	0.655
Feeling in control of your health	402	3.4 (1.0) [1–5]	3.5 (1.0) [1–5]	3.3 (1.0) [1–5]	0.244

* P value of Chi-square, ANOVA, or t-tests

### Reasons for using the app

Among the reasons for using MyChart, users reported convenience, ease of use, and the need to control their health. Most users who chose MyChart to access their health records (n = 415), declared to do so to track their health (307/415, 74%) or for convenience (262/415, 63%), ease of use (255/415, 61%), saving time (209/415, 50%), and to know more about their health (187/415, 45%). Among those who used MyChart to schedule in-person appointments (n = 283), the most frequently reported reasons were ease of use (196/283, 69%), convenience (183/283, 65%), and saving time (190/283, 67%). The most frequently mentioned reasons for using the messaging feature (n = 233) were also convenience (145/233, 62%), ease of use (139/233, 56%), and saving time (122/233, 52%). The complete list of reasons for using MyChart features is included in Supplementary Table 1.

The less used MyChart features were conducting remote visits (n = 391) and accessing the patient education section (n = 335). Most users did not use such features because they did not know these existed, were not interested, or preferred human interaction. The latter was the main reason for not scheduling remote visits (195/391, 50%). Lack of knowledge about the patient education section was the main reason for not using it (167/335, 50%). The complete list of reasons for not using MyChart features is included in Supplementary Table 2.

Based on the 5-point scale ranging from a considerable extent to a minimal extent, most participants considered MyChart to improve their ability to access health information (3.7 ± 1.0), providing better control of their health (3.4 ± 1.0), and enhance communication with their physician (3.0 ± 1.2).

### MyChart quality evaluation

Table 3 reports the average scores for the objective domains of engagement, functionality, aesthetics, and information quality, the total score, and the subjective quality score. According to uMARS completers (n = 200), the average objective quality score (total uMARS) was 3.8 ± 0.5. The highest rating domains were information quality (4.2 ± 0.5), followed by functionality (4.0 ± 0.7), aesthetics (3.9 ± 0.7), and engagement (3.3 ± 0.7). The average subjective quality score was 3.6 ± 0.7, positively associated with the uMARS total score (r = 0.505, P < 0.001). The uMARS total score was not significantly related to any demographic variables, app usage, or features. However, uMARS completers tended to be younger individuals who used more features especially remote visits, who were of lower socioeconomic status and had no chronic illnesses.

**Table 3:** Average values of app quality using the Mobile App Rating Scale (uMARS) (n = 200)

Variables	Mean (SD)
Engagement	3.3 (0.7)
Functionality	4.0 (0.7)
Aesthetics	3.9 (0.7)
Information quality	4.2 (0.5)
Total Score	3.8 (0.5)
Subjective quality	3.6 (0.7)

### Associations with the frequency of app use

The frequency of app use was significantly associated with some demographic and app quality variables, such as education, crowding index, perceived health status, chronic conditions, number of physician visits, subjective app quality, and number of features used (see Table 4).

Less frequent app use was reported among participants with higher education (Chi-square = 24.3, P < 0.001), high socioeconomic status (F(4,399) = 5.5, P < 0.001), higher perceived health status (Chi-square = 60.6, P < 0.001), and without a chronic illness (Chi-square = 13.1, P = 0.011). Those who used the app more frequently also reported a significantly higher number of physician visits before (F(4,186) = 3.8, P = 0.005), and after the pandemic (F(4,105.8) = 5.4, P < 0.001). Also, those who used the app more frequently perceived it as having higher subjective quality (F(4,195) = 7.6, P < 0.001), and were using more features (F(4,422) = 16.3, P < 0.001).

**Table 4:** Associations between frequency of app use and demographic variables

Variables	N	Frequency of app use, n (%)					P-value*
		More than once a week	Once a week	Once a month	Once every few months	Once a year	
		M (SD)					
Age	417	44.4 (16)	40.7 (15.8)	40.7 (15.4)	41.7 (15.5)	37.7 (16.8)	0.565
Crowding index	404	1.6 (0.6)	1.4 (0.6)	1.5 (0.6)	1.2 (0.5)	1.4 (0.6)	< 0.001
Total uMARS score	200	3.8 (0.6)	3.9 (0.6)	3.9 (0.5)	3.8 (0.5)	3.8 (0.5)	0.471
Subjective quality	200	4.2 (1.4)	3.9 (0.5)	3.6 (0.5)	3.4 (0.7)	3.1 (0.6)	< 0.001
Number of physician visits/year before the COVID-19 pandemic	417	3.1 (2.6)	3.8 (4.9)	3.4 (3.9)	2.4 (2.2)	1.5 (0.9)	0.005
Number of physician visits/year after the COVID-19 pandemic	410	3.7 (3.0)	4.3 (6.5)	3.1 (2.3)	2.2 (1.7)	1.4 (1.0)	< 0.001
		n(%)					
Gender	424						0.650
Female		26 (63.4)	34 (54.8)	77 (64.2)	117 (64.6)	14 (70.0)	
Male		15 (36.6)	28 (45.2)	43 (35.8)	64 (35.4)	6 (30.0)	
Education	422						< 0.001
Higher education		27 (65.9)	51 (82.3)	104 (87.4)	168 (93.3)	18 (90.0)	
Low education		14 (34.2)	11 (17.7)	15 (12.6)	12 (6.7)	2 (10.0)	
Area of primary residence	415						0.253
Beirut		24 (60.0)	33 (54.1)	75 (63.6)	119 (67.2)	9 (47.4)	
Bekaa		1 (2.5)	1 (1.6)	1 (0.8)	0 (0)	0 (0)	
Mount Lebanon		11 (27.5)	21 (34.4)	35 (29.7)	49 (27.7)	6 (31.6)	
North		4 (10.0)	4 (6.6)	3 (2.5)	5 (2.8)	3 (15.8)	
South		0 (0)	2 (3.3)	4 (3.4)	4 (2.3)	1 (5.3)	
Perceived health status	424						< 0.001
Poor		4 (9.8)	4 (6.6)	0 (0)	0 (0)	0 (0)	
Fair		8 (19.5)	4 (6.6)	14 (11.7)	13 (7.1)	0 (0)	
Good		21 (51.2)	33 (54.1)	48 (40.0)	67 (36.8)	4 (20.0)	
Very Good		5 (12.2)	17 (27.9)	47 (39.2)	75 (41.2)	12 (60.0)	
Excellent		3 (7.3)	3 (4.9)	11 (9.2)	27 (14.8)	4 (20.0)	
Chronic illnesses	419						0.011
Present		19 (46.3)	26 (43.3)	41 (34.7)	50 (27.6)	2 (10.5)	
Absent		22 (53.7)	34 (56.7)	77 (65.3)	131 (72.4)	17 (89.5)	
Number of features used	427	3.0 (1.0)	3.0 (0.9)	2.6 (0.9)	2.1 (1.0)	1.9 (1.1)	< 0.001

* P value of Chi-square, ANOVA, or t-tests

## Discussion

### Principal findings

This study evaluated a patient portal app usage (MyChart), its features and reasons for usage, and its perceived quality according to current app users. This study also explored the association among app usage, patient characteristics, and perceived app quality.

In our study, most participants used the app once a month or less, and only a quarter were weekly or daily users. The frequency of patient portal usage is similar to a sample of patient portal users in the US, whereby more than 78% of users used the portal at least once per month [54]. Regarding features used, we expected a high frequency of remote visits scheduled through MyChart during the pandemic, as reported in other studies in the Middle East [55, 56].

Nevertheless, only about 7.8% of the respondents used remote visits. Similar rates of remote visits were reported in a large-scale study conducted on 197 clinic centers in the US, where 13% had scheduled remote visits instead of in-person visits [55, 56]. Using remote visits was higher among the subsample of uMARS completers (n = 200) who also used more features than their counterparts. This indicates that uMARS completers were also tech-savvy users. This might be because remote visits were delivered through another app, Webex, which is the preferred remote meetings service in use at our institution. A patient would have needed to schedule an appointment with MyChart and then use the Webex app to complete the visit, increasing the difficulty among those less tech-savvy. This may have contributed to the low usage. Another reason might be the need for human interaction that some participants expressed. Finally, the preference for in-person appointments may stem from cultural or legal concerns, namely patients’ apprehension surrounding privacy and their reluctance to change the conventional way of seeking medical advice [57, 58]. Recent reports found that around 84% of the Lebanese population had access to the internet in 2020 [38], and 86% of the population owned smartphones in 2019 [39]. Therefore, despite the increased usage of remote visits worldwide during the pandemic, our study’s relatively low engagement with remote visits can be attributed to cultural rather than logistical factors. Future research is needed to understand the reasons behind the reluctance towards remote visits within the Arab context and recommended approaches to encourage tele-visits.

### App quality

The MyChart app was rated quite positively, reaching an overall score of 3.8/5 according to the Mobile App Rating Scale (uMARS). This is consistent with other studies investigating the quality of health apps using the same instrument [59, 60] or the quality of patient portals using other usability scales [61–63]. The least-scoring app domain was engagement (3.3/5). This might be explained by the fact that MyChart is a health portal that provides users with personal results and objective health information, rather than entertaining and attracting them. Engagement could be incorporated in the patient education section as most participants did not use the patient education section. A study conducted among patients in the same institution before the implementation of the portal has shown that perceived usefulness had more predictive value than perceived ease of use on the intention to use the patient portal [33]. Although the frequency of app use was not significantly associated with the total uMARS score, it was positively associated with the subjective quality score. Also, uMARS completers were more likely to be young and used more features than non-completers, suggesting that they perceived the app better and increased their use. A discrepancy between objective and subjective MARS scores is reported in the literature [50, 55, 56], suggesting that patients who used the app more frequently may still perceive it as valuable and useful despite objectively considering it an average asset. Furthermore, the usability of MyChart app can be further assessed using usability tests in research design to identify weaknesses of the app and provide recommendations accordingly. This has been done in the literature in a number of studies evaluating the usability of apps using qualitative data generated by interviewing participants to get their feedback on the specific aspects of the app [64, 65].

### Associations with the frequency of app use

In our study, the app usage frequency was negatively related to education, the crowding index (a proxy of socioeconomic status), and perceived health status. App usage was positively associated with having chronic conditions, number of physician visits, number of features used, and subjective app quality. Overall, healthier and more affluent participants used the app less frequently, as they might have needed it less. This finding might be due to higher levels of health literacy (and digital health literacy) among highly educated individuals, who tend to use digital technology for health information seeking, as reported in a recent study conducted among Lebanese internet users [30].

The observed associations should be considered bearing in mind the characteristics of the sample, which includes predominantly females of high socioeconomic status and high education. In addition, we recruited a sample including a broader range of ages compared to other studies in which most patient portal users were younger [54, 66, 67]. This might explain why we did not observe significant associations between app usage and age or gender. Regarding gender differences, the literature is mixed, with some studies reporting no association [68, 69], and some positive associations [70–72], depending on the samples recruited. Regarding education and socioeconomic status, our sample was also quite homogeneous and included a relatively high number of participants with a high level of education and socioeconomic status. Yet, app use was significantly lower among those with high education and socioeconomic status. The crowding index in our sample was 1.4, which indicates that our sample is skewed towards a higher socioeconomic status. Hence, the observed association with frequency is questionable and cannot be generalized given that our sample is predominantly of high socioeconomic status. Several studies showed that patients with a low socioeconomic status tend to use patient portals less frequently. This was attributed to a lower likelihood of internet access and a lower education level [73–77]. Further studies should investigate the factors affecting patient portals’ usage patterns, especially among those less educated, less affluent individuals.

### Limitations and directions for future research

There are some biases present in our study that would certainly affect the generalizability of the results. One example is self-selection bias, as the MyChart app is only available in English. While the official language of Lebanon is Arabic, English and French are spoken among highly educated groups. This might explain why our sample included highly educated respondents. Furthermore, the online survey was sent via the MyChart application only to users who had previously agreed to be contacted for studies conducted by the hospital. Therefore, many users who had not opted for this feature upon registering were not contacted. Both issues may be addressed by adopting data collection strategies that yield a more diverse sample such as stratified sampling with strata including male users and individuals with limited education and health and digital literacies. Moreover, although anonymity and confidentiality were firmly assured, respondents might have been influenced by social desirability bias. They were probably aware that patient portals were desirable for the hospital leadership and responded more positively.

As is the case with most survey studies, the self-reporting nature of a survey influences the objectivity of the study whereby participants may tend to respond the way they perceive would be more desirable, thereby exaggerating their use of certain features. This can be addressed in further studies by using more objective measures such as data from the IT center regarding the specific use of certain features of the portal. This includes number of clicks on a certain link or time spent on a webpage which may reflect better the usage of the portal. Also, recall bias could have been present, as patients were asked to recall past usage and some respondents might have forgotten features they had used a long time prior to the study. The best way to tackle this bias is to use a prospective design in future studies where the usage of the app features by the recruited participants is monitored by the researchers making the process more objective.

This study adds to the literature that the features and patient demographics may be more important than the actual aesthetic properties of the app. Therefore, app developers should prioritize developing the existing features and making them more accessible. Moreover, healthcare providers can play a role in enhancing usability of the app mainly by raising awareness about the different features available, especially the ones used less frequently such as remote visits. More research is warranted to optimize the utilization of patient portals and to improve healthcare delivery in the Arab world, possibly including randomized controlled trial designs and longitudinal studies to minimize selection and response biases.

## Conclusion

In our study of patient portals accessed through the MyChart mobile app, most users utilized the app once a month to once every few months. Overall, MyChart users found the app easy to use, and informative, yet they utilized a limited number of features. The frequency of app use was inversely associated with high education, socioeconomic status, and perceived health status, and positively related with having chronic conditions, number of visits, and perceived subjective app quality. Yet, most of the sample included females, with high education and socioeconomic status who were relatively healthy.

### Electronic supplementary material

Below is the link to the electronic supplementary material.

Supplementary Material 1

Supplementary Material 2

Supplementary Material 3

## Data Availability

The dataset analyzed in the study is available from the corresponding author upon reasonable request.
