# Supplementary material for: Utilization of patient portals: a cross-sectional study investigating associations with mobile app quality
Source: BMC Med Inform Decis Mak. 2023 Sep 5;23:177. doi: 10.1186/s12911-023-02252-x (PMC10481578; doi:10.1186/s12911-023-02252-x)
Supplement: Supplementary file 1 — Supplementary Material 1 [file 12911_2023_2252_MOESM1_ESM.docx]

Supplementary Table 1: Reasons for using app features

| **Reasons for using** | **Scheduling in-person appointments (n=283, 66.6%)** | **Remote visits (n= 33, 7.8%)** | **Messaging**  **(n= 233, 55.6%)** | **Personal health record**  **(n=415, 98.3%)** | **Patient education**  **(n=82, 19.2%)** |
| --- | --- | --- | --- | --- | --- |
| It was convenient | 183 (64.7) | 9 (27.3) | 145 (62.2) | 262 (63.1) | 18 (22) |
| It was easy to use | 196 (69.3) | 11 (33.3) | 130 (55.8) | 255 (61.4) | 21 (25.6) |
| It allowed me to save time | 190 (67.1) | 10 (30.3) | 122 (52.4) | 209 (50.4) | 7 (8.5) |
| It allowed me to save money | 28 (9.9) | 2 (6.1) | 22 (9.4) | 34 (8.2) | 3 (3.7) |
| It avoids unnecessary human action (asking someone to book an appointment on my behalf) | 122 (43.1) | 0 (0.0) | 0 (0.0) | 0 (0.0) | 0 (0.0) |
| The appointments usually sync directly to my phone’s calendar | 56 (19.8) | 4 (12.1) | 0 (0.0) | 0 (0.0) | 0 (0.0) |
| It was the only way to get an appointment with my healthcare provider | 0 (0.0) | 9 (27.3) | 0 (0.0) | 0 (0.0) | 0 (0.0) |
| It was safer option in the pandemic | 0 (0.0) | 14 (42.4) | 0 (0.0) | 0 (0.0) | 0 (0.0) |
| It was the only way to reach out to my healthcare provider | 0 (0.0) | 0 (0.0) | 84 (36.1) | 0 (0.0) | 0 (0.0) |
| I wanted to track my health | 0 (0.0) | 0 (0.0) | 0 (0.0) | 307 (74) | 0 (0.0) |
| I wanted to know more about my health | 0 (0.0) | 0 (0.0) | 0 (0.0) | 187 (45.1) | 69 (84.1) |
| I was just curious | 0 (0.0) | 0 (0.0) | 0 (0.0) | 0 (0.0) | 36 (43.9) |
| Note: the sum of reasons >100% as the question was multiple-choice | | | | | |
