# Supplementary material for: Utilization of patient portals: a cross-sectional study investigating associations with mobile app quality
Source: BMC Med Inform Decis Mak. 2023 Sep 5;23:177. doi: 10.1186/s12911-023-02252-x (PMC10481578; doi:10.1186/s12911-023-02252-x)
Supplement: Supplementary file 2 — Supplementary Material 2 [file 12911_2023_2252_MOESM2_ESM.docx]

Supplementary Table 2: Reasons for not using app features

| **Reasons for not using** | **Scheduling appointments n=142**  **(33.4%)** | **Remote visits**  **n=391 (92.2%)** | **Messaging healthcare providers n=186 (44.4%)** | **Health records/test results**  **n=7 (1.7%)** | **Patient education section**  **n=335 (80.3%)** |
| --- | --- | --- | --- | --- | --- |
| I didn’t know it existed | 22 (15.5) | 92 (23.5) | 48 (25.8) | 0 (0.0) | 167 (49.9) |
| I was not interested; I didn’t feel I needed it | 19 (13.4) | 112 (28.6) | 67 (36.0) | 3 (42.9) | 111 (33.1) |
| I found it hard to use | 5 (3.5) | 6 (1.5) | 4 (2.2) | 0 (0.0) | 2 (0.6) |
| I did not know how to use the feature | 9 (6.3) | 16 (4.1) | 0 (0.0) | 0 (0.0) | 0 (0.0) |
| I usually have internet connectivity issues | 1 (0.7) | 18 (4.6) | 2 (1.1) | 0 (0.0) | 7 (2.1) |
| I usually prefer human interaction | 75 (52.8) | 195 (49.9) | 60 (32.3) | 0 (0.0) | 0 (0.0) |
| I usually prefer calling the doctor over the phone | 0 (0.0) | 0 (0.0) | 16 (8.6) | 0 (0.0) | 0 (0.0) |
| I usually prefer using text messages or another app for messaging (e.g., WhatsApp) | 0 (0.0) | 0 (0.0) | 9 (4.8) | 0 (0.0) | 0 (0.0) |
| I don’t think the physician’s replies will be clear | 0 (0.0) | 0 (0.0) | 9 (4.8) | 0 (0.0) | 0 (0.0) |
| Appointments do not usually sync with my phone’s calendar | 2 (1.4) | 1 (0.3) | 0 (0.0) | 0 (0.0) | 0 (0.0) |
| The department I am seeking does not offer appointments via the application | 7 (4.9) | 14 (3.6) | 0 (0.0) | 0 (0.0) | 0 (0.0) |
| I previously tried the feature and was not satisfied with it | 1 (0.7) | 2 (0.5) | 0 (0.0) | 0 (0.0) | 3 (0.9) |
| The virtual visit is not conducted via MyAUBHealth application. Navigating between two applications is not ideal | 0 (0.0) | 1 (0.3) | 0 (0.0) | 0 (0.0) | 0 (0.0) |
| I am concerned about my privacy | 0 (0.0) | 8 (2) | 1 (0.5) | 0 (0.0) | 0 (0.0) |
| I do not trust online payment methods | 0 (0.0) | 8 (2) | 0 (0.0) | 0 (0.0) | 0 (0.0) |
| I did not know how to find the feature on the app | 0 (0.0) | 0 (0.0) | 0 (0.0) | 1 (14.3) | 19 (5.7) |
| I prefer having all the information as a hardcopy | 0 (0.0) | 0 (0.0) | 0 (0.0) | 1 (14.3) | 0 (0.0) |
| I prefer using other resources | 0 (0.0) | 0 (0.0) | 0 (0.0) | 0 (0.0) | 54 (16.1) |
| Note: Sum of reasons >100% as the question was multiple-choice | | | | | |
