# Supplementary material for: Utilization of patient portals: a cross-sectional study investigating associations with mobile app quality
Source: BMC Med Inform Decis Mak. 2023 Sep 5;23:177. doi: 10.1186/s12911-023-02252-x (PMC10481578; doi:10.1186/s12911-023-02252-x)
Supplement: Supplementary file 3 — Supplementary Material 3 [file 12911_2023_2252_MOESM3_ESM.pdf]

A) Background

1. What is your age? ____
2. What is your sex?
    1. Male
    2. Female
3. What is the highest educational level that you attained?
    1. No formal schooling
    2. Elementary
    3. Middle school
    4. High school
    5. Higher education

4. Where is the area of your primary residence?
    1. Beirut
    2. Bekaa
    3. Mount Lebanon
    4. Nabatieh
    5. North
    6. South
5. How many people including yourself live in your primary residence? (Include all members: Family, non-family, maids/helpers. Exclude newborn infants)

____

6. How many bedrooms does your primary residence have?

____

7. How is your health in general?
    1. Poor
    2. Fair
    3. Good
    4. Very good
    5. Excellent

8. Before the Covid-19 pandemic, how many times did you visit a doctor/physician on average per year?

____

9. After the onset of the Covid-19 pandemic, how many times did you visit a doctor/physician on average per year?

____

10. Do you live with any chronic illness (e.g., diabetes, chronic kidney disease, asthma, etc.)?
    1. Yes
    2. No

B) Questions about app usage

Q1) On average, how many times did you use MyChart app on your phone in the past year?

1. More than once a week
2. Once a week
3. Once a month
4. Once every few months
5. Once a year

Q2) Have you ever used MyChart app for scheduling in-person clinic visits (appointments)?

- ❑ Yes
- ❑ No

Q2a) If the responder answers Yes --> Why have you used the scheduling feature? Select any that apply.

- ❑ It was convenient
- ❑ It was easy to use
- ❑ It allowed me to save time
- ❑ It allowed me to save money
- ❑ It avoids unnecessary human action (asking someone to book an appointment on my behalf)
- ❑ The appointments usually sync directly to my phone's calendar
- ❑ Other (please specify)____________

Q2b) If the responder answers No --> Why haven't you used the scheduling feature? Select all that apply.

- ❑ I didn't know it existed
- ❑ I was not interested; I didn't feel I needed it
- ❑ I found it hard to use
- ❑ I did not know how to use the feature
- ❑ I usually have internet connectivity issues
- ❑ I usually prefer human interaction to book an appointment
- ❑ Appointments do not usually sync with my phone's calendar
- ❑ The department I am seeking does not offer appointments via the application
- ❑ I previously tried the feature and was not satisfied with it
- ❑ Other (please specify) _____

Q3) Have you ever used MyChart app for scheduling remote video visits (telemedicine)?

- ❑ Yes
- ❑ No

Q3a) If the responder answers Yes --> Why have you used the remote video visit? Select all that apply.

- ❑ It was the only way to get an appointment with my healthcare provider
- ❑ It is convenient
- ❑ It's easy to use
- ❑ It saves time
- ❑ It saves money
- ❑ The appointments sync directly to my phone's calendar
- ❑ It was safer option in the pandemic
- ❑ Other (please specify)______

Q3b) If the responder answers No --> Why haven't you used the video visit feature? Select all that apply.

- ❑ I didn't know it existed
- ❑ I am not interested, I don't need it
- ❑ I find it hard to use
- ❑ I do not know how to use the feature
- ❑ I have internet connectivity issues
- ❑ I prefer seeing the doctor in person
- ❑ Appointments do not sync with my phone's calendar
- ❑ The department I am seeking does not offer telemedicine appointments
- ❑ I previously tried the feature and was not satisfied with it
- ❑ The virtual visit is not conducted via MyAUBHealth application. Navigating between two applications is not ideal.
- ❑ I am concerned about my privacy
- ❑ I do not trust online payment methods
- ❑ Other _____

Q4) Have you ever used MyChart for messaging your healthcare provider?

- ❑ Yes
- ❑ No

Q4a) If the responder answers Yes --> Why have you used the messaging feature? Select all that apply.

- ❑ It was the only way to reach out to my healthcare provider
- ❑ It was convenient
- ❑ It was easy to use
- ❑ It allowed me to save time
- ❑ It allowed me to save money
- ❑ Other (please specify)______

Q4b) If the responder answers No --> Why haven't you used the messaging feature? Select all that apply.

- ❑ I didn't know it existed
- ❑ I was not interested; I didn't feel the need for it
- ❑ I found it hard to use
- ❑ I usually have internet connectivity issues
- ❑ I usually prefer talking with a doctor in person
- ❑ I usually prefer calling the doctor over the phone
- ❑ I usually prefer using text messages or another app for messaging (e.g., WhatsApp)
- ❑ I previously tried the feature and was not satisfied with it
- ❑ I am concerned about privacy
- ❑ I don't think the physician's replies will be clear
- ❑ Other _____

Q5) Have you ever used MyChart app for accessing your personal health record, to view test results and/or medications?

- ❑ Yes
- ❑ No

Q5a) If responder answers Yes --> Why have you used the personal health record feature to view test results and/or medications? Select all that apply.

- ❑ I wanted to track my health
- ❑ I wanted to know more about my health

❑ It was convenient

❑ It was easy to use

❑ It allowed me to save time

❑ It allowed me to save money

❑ Other (please specify)______

Q5b) If responder answers No --> Why haven't you used your personal health record to view test results and/or medications? Select all that apply.

❑ I didn't know it existed

❑ I was not interested; I didn't feel the need for it

❑ I found it hard to use

❑ I did not know how to find my personal health record on the app

❑ I usually have internet connectivity issues

❑ I previously tried the feature and was not satisfied with it
❑ I prefer having all the information as a hardcopy
❑ Other ______

Q6) Have you used MyChart app for learning more about diseases or for managing your diseases (Patient education section)?

❑ Yes
❑ No

Q6a) If responder answers Yes --> Why did you use the patient education section? Select all that apply.

❑ I wanted to learn more about my condition
❑ I wanted to learn more about my health
❑ I was just curious
❑ It was convenient
❑ The section was user friendly
❑ It allowed me to save time
❑ It allowed me to saves money
❑ Other ______

Q6b) If responder answers No --> Why haven't you used the patient education section? Select all that apply.

- ❑ I didn't know it existed
- ❑ I was not interested; I didn't feel the need for it
- ❑ I found it hard to use
- ❑ I did not know how to find related patient education on the app
- ❑ I usually have internet connectivity issues
- ❑ I previously tried the feature and was not satisfied with it
- ❑ I prefer using other resources
- ❑ Other _____

Q7) To what extent do you think MyChart app improved your access to health information?

1. To a very small extent
2. To a small extent
3. To a moderate extent
4. To a large extent
5. To a very large extent

Q8) To what extent do you believe that MyChart app improved your communication with the physician?

1. To a very small extent
2. To a small extent
3. To a moderate extent
4. To a large extent
5. To a very large extent

Q9) To what extent did MyChart app provided you with control of your health?

1. To a very small extent
2. To a small extent
3. To a moderate extent
4. To a large extent
5. To a very large extent

Q10) Did you use any other features of the app? If yes, please specify

1. Yes , please specify: ___________________________
2. No

C) uMARS

SECTION A

Engagement – fun, interesting, customisable, interactive, has prompts (e.g. sends alerts, messages, reminders, feedback, enables sharing)

1. **Entertainment: Is the app fun/entertaining to use? Does it have components that make it more fun than other similar apps?**

1 Dull, not fun or entertaining at all
2 Mostly boring
3 OK, fun enough to entertain user for a brief time (< 5 minutes)
4 Moderately fun and entertaining, would entertain user for some time (5-10 minutes total)
5 Highly entertaining and fun, would stimulate repeat use

2. **Interest: Is the app interesting to use? Does it present its information in an interesting way compared to other similar apps?**

1 Not interesting at all
2 Mostly uninteresting
3 OK, neither interesting nor uninteresting; would engage user for a brief time (< 5 minutes)
4 Moderately interesting; would engage user for some time (5-10 minutes total)
5 Very interesting, would engage user in repeat use

3. **Customisation: Does it allow you to customise the settings and preferences that you would like to (e.g. sound, content and notifications)?**

1 Does not allow any customisation or requires setting to be input every time
2 Allows little customisation and that limits app's functions
3 Basic customisation to function adequately
4 Allows numerous options for customisation
5 Allows complete tailoring the user's characteristics/preferences, remembers all settings

4. **Interactivity: Does it allow user input, provide feedback, contain prompts (reminders, sharing options, notifications, etc.)?**

1 No interactive features and/or no response to user input
2 Some, but not enough interactive features which limits app's functions

3 Basic interactive features to function adequately
4 Offers a variety of interactive features, feedback and user input options
5 Very high level of responsiveness through interactive features, feedback and user input options

5. **Target group: Is the app content (visuals, language, design) appropriate for the target audience?**

1 Completely inappropriate, unclear or confusing
2 Mostly inappropriate, unclear or confusing
3 Acceptable but not specifically designed for the target audience. May be inappropriate/ unclear/confusing at times
4 Designed for the target audience, with minor issues
5 Designed specifically for the target audience, no issues found

SECTION B

Functionality – app functioning, easy to learn, navigation, flow logic, and gestural design of app

6. **Performance: How accurately/fast do the app features (functions) and components (buttons/menus) work?**

1 App is broken; no/insufficient/inaccurate response (e.g. crashes/bugs/broken features, etc.)
2 Some functions work, but lagging or contains major technical problems
3 App works overall. Some technical problems need fixing, or is slow at times
4 Mostly functional with minor/negligible problems
5 Perfect/timely response; no technical bugs found, or contains a 'loading time left' indicator (if relevant)

7. **Ease of use: How easy is it to learn how to use the app; how clear are the menu labels, icons and instructions?**

1 No/limited instructions; menu labels, icons are confusing; complicated
2 Takes a lot of time or effort
3 Takes some time or effort
4 Easy to learn (or has clear instructions)
5 Able to use app immediately; intuitive; simple (no instructions needed)

8. **Navigation: Does moving between screens make sense; Does app have all necessary links between screens?**

1 No logical connection between screens at all /navigation is difficult
2 Understandable after a lot of time/effort
3 Understandable after some time/effort
4 Easy to understand/navigate
5 Perfectly logical, easy, clear and intuitive screen flow throughout, and/or has shortcuts

9. **Gestural design: Do taps/swipes/pinches/scrolls make sense? Are they consistent across all components/screens?**

1 Completely inconsistent/confusing
2 Often inconsistent/confusing
3 OK with some inconsistencies/confusing elements
4 Mostly consistent/intuitive with negligible problems
5 Perfectly consistent and intuitive

SECTION C

Aesthetics – graphic design, overall visual appeal, colour scheme, and stylistic consistency

10. **Layout: Is arrangement and size of buttons, icons, menus and content on the screen appropriate?**

1 Very bad design, cluttered, some options impossible to select, locate, see or read
2 Bad design, random, unclear, some options difficult to select/locate/see/read
3 Satisfactory, few problems with selecting/locating/seeing/reading items
4 Mostly clear, able to select/locate/see/read items
5 Professional, simple, clear, orderly, logically organised

11. **Graphics: How high is the quality/resolution of graphics used for buttons, icons, menus and content?**

1 Graphics appear amateur, very poor visual design - disproportionate, stylistically inconsistent
2 Low quality/low resolution graphics; low quality visual design – disproportionate
3 Moderate quality graphics and visual design (generally consistent in style)
4 High quality/resolution graphics and visual design – mostly proportionate, consistent in style
5 Very high quality/resolution graphics and visual design - proportionate, consistent in style throughout

12. **Visual appeal: How good does the app look?**

1 Ugly, unpleasant to look at, poorly designed, clashing, mismatched colours
2 Bad – poorly designed, bad use of colour, visually boring
3 OK – average, neither pleasant, nor unpleasant
4 Pleasant – seamless graphics – consistent and professionally designed
5 Beautiful – very attractive, memorable, stands out; use of colour enhances app features/menus

SECTION D

Information – Contains high quality information (e.g. text, feedback, measures, references) from a credible source

13. **Quality of information: Is app content correct, well written, and relevant to the goal/topic of the app?**

N/A There is no information within the app
1 Irrelevant/inappropriate/incoherent/incorrect
2 Poor. Barely relevant/appropriate/coherent/may be incorrect
3 Moderately relevant/appropriate/coherent/and appears correct
4 Relevant/appropriate/coherent/correct
5 Highly relevant, appropriate, coherent, and correct

14. **Quantity of information: Is the information within the app comprehensive but concise?**

N/A There is no information within the app
1 Minimal or overwhelming
2 Insufficient or possibly overwhelming
3 OK but not comprehensive or concise
4 Offers a broad range of information, has some gaps or unnecessary detail; or has no links to more information and resources
5 Comprehensive and concise; contains links to more information and resources

15. **Visual information: Is visual explanation of concepts – through charts/graphs/images/videos, etc. – clear, logical, correct?**

N/A There is no visual information within the app (e.g. it only contains audio, or text)
1 Completely unclear/confusing/wrong or necessary but missing
2 Mostly unclear/confusing/wrong
3 OK but often unclear/confusing/wrong
4 Mostly clear/logical/correct with negligible issues
5 Perfectly clear/logical/correct

16. **Credibility of source: does the information within the app seem to come from a credible source?**

N/A There is no information within the app

1 Suspicious source

2 Lacks credibility

3 Not suspicious but legitimacy of source is unclear

4 Possibly comes from a legitimate source

5 Definitely comes from a legitimate/specialised source

App subjective quality

SECTION E

---

17. **Would you recommend this app to people who might benefit from it?**

1	Not at all	I would not recommend this app to anyone
2		There are very few people I would recommend this app to
3	Maybe	There are several people I would recommend this app to
4		There are many people I would recommend this app to
5	Definitely	I would recommend this app to everyone

18. **How many times do you think you would use this app in the next 12 months if it was relevant to you?**

1 None

2 1-2

3 3-10

4 10-50

5 >50

19. **Would you pay for this app?**

1 Definitely not

2

3

4

5 Definitely yes

20. **What is your overall (star) rating of the app?**

1	★	One of the worst apps I've used
2	★★	
3	★★★	Average
4	★★★★	
5	★★★★★	One of the best apps I've used

21. Any further comments on the app: ____
